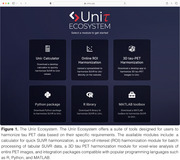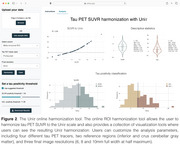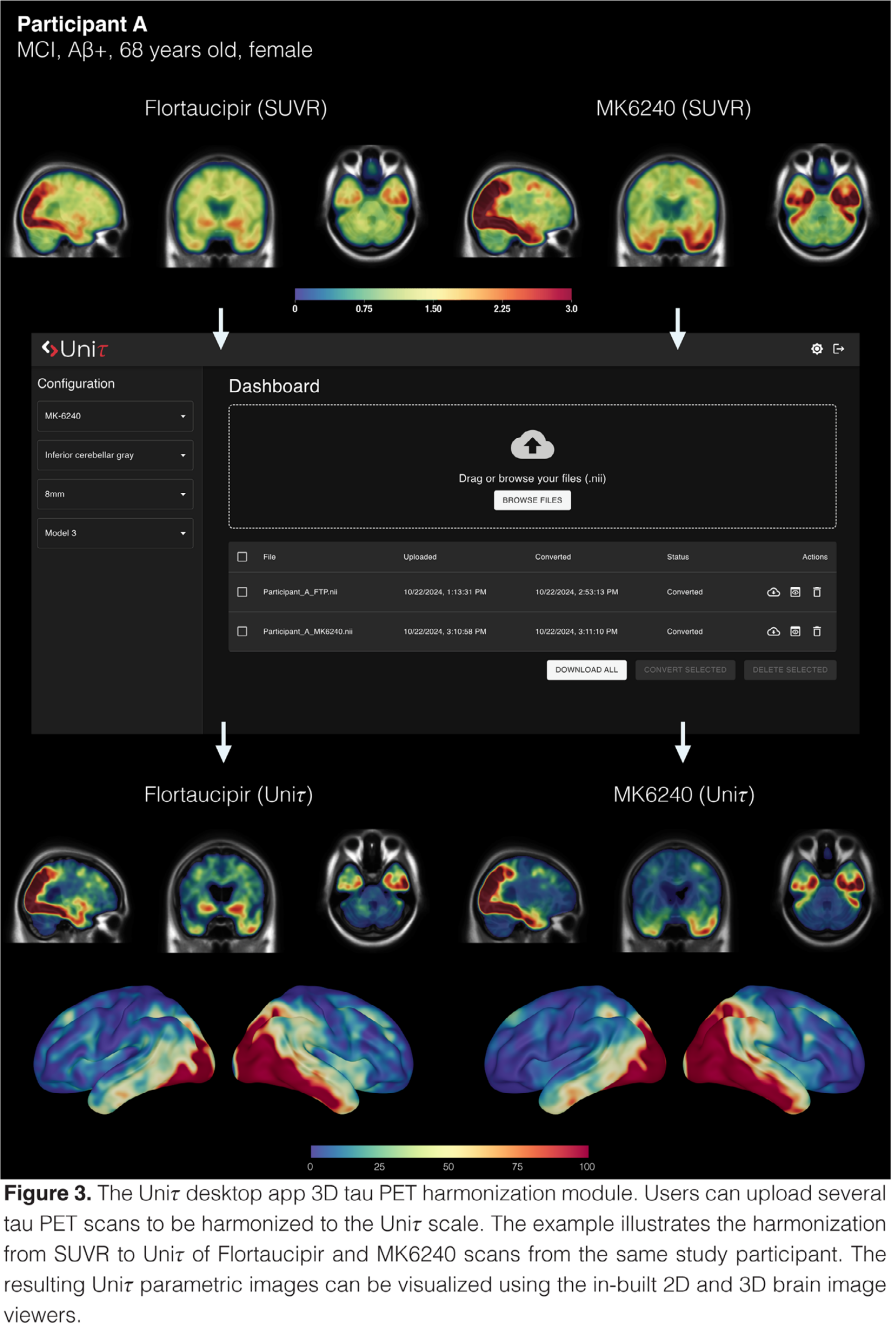# User‐friendly tools for tau PET harmonization in clinical trials: The Uniτ Ecosystem

**DOI:** 10.1002/alz70859_107201

**Published:** 2025-12-26

**Authors:** Guilherme Povala, Guilherme Bauer‐Negrini, Bruna Bellaver, Pamela C.L. Ferreira, Livia Amaral, Firoza Z Lussier, Dana L Tudorascu, Quentin Finn, Joseph C. Masdeu, David N. Soleimani‐Meigooni, Juan Fortea, Val J Lowe, Hwamee Oh, Belen Pascual, Brian A. Gordon, Pedro Rosa‐Neto, Suzanne L. Baker, Tharick A Pascoal

**Affiliations:** ^1^ University of Pittsburgh, Pittsburgh, PA USA; ^2^ Houston Methodist Research Institute, Houston, TX USA; ^3^ University of California, San Francisco, San Francisco, CA USA; ^4^ Sant Pau Memory Unit, Hospital de la Santa Creu i Sant Pau, Biomedical Research Institute Sant Pau, Barcelona Spain; ^5^ Mayo Clinic, Rochester, MN USA; ^6^ Brown University, Providence, RI USA; ^7^ Washington University in St. Louis, St. Louis, MO USA; ^8^ McGill University, Montreal, QC Canada; ^9^ Lawrence Berkeley National Laboratory, Berkeley, CA USA

## Abstract

**Background:**

HEAD is a longitudinal, multi‐site, non‐randomized study aiming to harmonize different tau PET tracers onto a common scale for enrichment and monitoring of anti‐tau clinical trials. To achieve this, the Uniτ scale is being developed using data from participants scanned head‐to‐head with Flortaucipir and MK6240, with a subset also scanned with PI2620 and RO948. However, access to the Uniτ scale has been limited to the study community and collaborators. To address this limitation, we have developed the Uniτ Ecosystem – a set of tools for tau PET harmonization designed to support research and clinical trials (www.unitau.app).

**Method:**

The Uniτ Ecosystem was developed for multiple operating systems (Web/macOS/Windows/Ubuntu) (Figure 1) and currently include four tools. The Uniτ harmonization is an online ROI harmonization module that allows the user to harmonize SUVRs from spreadsheets. The Uniτ packages (R, Python and MATLAB) facilitates the transformation of SUVR values to the Uniτ scale using the most used programming languages in research settings. The Uniτ calculator app allows rapid and convenient harmonization. The Uniτ desktop app is a 3D harmonization module for voxel‐wise harmonization. The Uniτ parameters were calculated using a training set of 200 participants with head‐to‐head MK6240 and Flortaucipir, and 90 individuals with additional PI2620 and RO948. Researchers can use their own datasets and convert tau PET data to the Uniτ scale.

**Result:**

The online ROI harmonization module allows users to harmonize tau PET SUVRs uploaded from spreadsheets and offers interactive visualizations such as scatter plots and histograms for tau positivity thresholds (Figure 2). The calculator app enables users to harmonize single SUVR values effortlessly. The desktop application for 3D tau PET harmonization leverages the functionalities of the previous modules to harmonize entire 3D images, eliminating the need for predefined ROIs. Additionally, this module provides visualization tools for the parametric Uniτ images in 2D and 3D (Figure 3). Uniτ tools never store any data on any external servers.

**Conclusion:**

The Uniτ Ecosystem represents a significant advance in tau PET harmonization, providing a user‐friendly platform to harmonize and visualize tau PET data for research and clinical trials.